# The Pathological Aspect

**Published:** 1936

**Authors:** Arthur L. Taylor

**Affiliations:** Pathologist, Bacteriologist, and Clinical Pathologist, Bristol General Hospital


### CARCINOMA OF THE BRONCHUS.

### The Pathological Aspect.

By ARTHUR L. TAYLOR, M.D.,

*Pathologist, Bacteriologist, and Clinical Pathologist, Bristol General Hospital.*

Incidence.—The following observations are based on a study of cases of bronchial carcinoma encountered in the Pathological Department of the General Hospital during the last eight years. In this period 32 cases have come to post-mortem, a fact which testifies to the very considerable frequency of these tumours. Similar large numbers are being reported from practically all the large hospitals in Europe and America, and it is believed in many quarters that lung cancer is on the increase. Whether the reported increase is real or not is a much debated question which is still far from settled. It is obvious that nowadays closer attention is being paid to the subject, and it may well be that more careful observation and improved methods of modern diagnosis account for the greater number of reported cases.

An analysis of the post-mortem records for the last eight years shows that bronchial carcinoma is second only to gastro-intestinal cancer in frequency, comprising 2·35 per cent. of all autopsies, and no less than 13·6 per cent. of all malignant deaths. Its incidence compared with that of other primary growths is seen in the following table :—

Table I.

Eight years, 1928 to 1935. Total post-mortems (excluding violent death) .. .. .. .. .. .. 1,360
Total malignant cases .. .. .. .. .. .. 236

| Primary Site. | No. | Percentage of all post-mortems. | Percentage of all malignant post-mortems. |
|---|---|---|---|
| Stomach .. .. .. | 41 | 3·01 | 17·4 |
| Intestine .. .. .. | 38 | 2·79 | 16·1 |
| Lung .. .. .. | 32 | 2·35 | 13·6 |
| Genito-urinary .. | 29 | 2·13 | 12·3 |
| Brain .. .. .. | 20 | 1·47 | 8·5 |
| Œsophagus .. .. | 19 | 1·40 | 8·1 |
| Other organs .. .. | 9 or less. | 0·70 or less. | 4 or less. |

Bronchial carcinoma is much commoner in men than in women; of our 32 cases 26 were males. The age incidence corresponds well with that of carcinoma generally: the average age in males was 49 years, in females 52 years, with an average for the whole group of just under 50 years. The youngest cases were males of 24 and 26, and the eldest 67 years. No special occupational incidence was observed in the 27 cases where this was known.

Morbid Anatomy.—In over 90 per cent. of cases lung carcinoma is of hilar origin, commencing in the mucosa of one of the large bronchi and spreading thence into the lung tissue and rapidly invading the bronchial lymph glands. All but one of our series were of this type. The tumour is whitish in colour and generally of firm consistency; if it is large it may show central cavitation from necrosis. In many cases (16 of our series) the growth extended widely into the mediastinum, infiltrating the roots of the great vessels and even penetrating the heart wall. The appearance thus presented may suggest a primary mediastinal growth, but careful dissection along the main bronchi reveals an ulcerated patch of mucosa sometimes surprisingly small, which indicates with certainty the true site of origin. Right and left lungs appear to suffer with almost equal frequency; in our cases 14 occurred on the right side and 17 on the left. The main bronchi were affected 9 times, the upper and middle 10 times and the lower 12. The condition of the lungs beyond the growth largely depends on two factors: the degree of bronchial stenosis, and the presence or otherwise of secondary infection. Thus the emphysema which occurs in the early stage of partial occlusion is replaced by massive collapse when the obstruction becomes complete. An added infection is almost constantly present in the later stages, and at post-mortem one commonly finds broncho-pneumonia, abscess formation and even gangrene of the obstructed lung tissue. This occurred in 20 cases; pleural effusion or empyema was found less frequently, in only 6 cases.

HISTOLOGY. — The microscopic classification of bronchial carcinoma is often a matter of difficulty owing to the great variety of cell types encountered, even in one and the same tumour. Three main types, however, may be described.

1. The *oatcell* type.—This, in our experience the commonest variety, consists of small undifferentiated round oval or pleomorphic cells possessing only scanty stroma and closely resembling sarcomatous tissue. It is this resemblance which has led to many diagnostic errors in the past; our present-day recognition of the true nature of these tumours helps largely to account for the alleged increase in bronchial carcinoma generally. 60 per cent. of our cases were of this type.

2. The *columnar-cell* type.—Histologically this is a true adeno-carcinoma, sometimes producing mucin and frequently showing a papilliform arrangement; occasionally the cells are grouped in solid alveolar formation.

3. The *squamous* type.—This is a rarer form, consisting of squamous epithelium usually of an undifferentiated type, in which horn production is not prominent, although cell nests may occur. It probably arises from an area of squamous metaplasia following chronic inflammatory lesions, since squamous epithelium is not a normal constituent of bronchial mucosa. Only three of our cases were of this type.

*Metastases.*—In addition to the almost invariable involvement of the tracheo-bronchial lymph glands, metastases from bronchial carcinoma are generally numerous and wide-spread. The relative frequency of secondary deposits in our 32 cases was as follows :—

TABLE II.

| | | |
|---|---|---|
| Tracheo-bronchial lymph glands .. | 31 | cases. |
| Abdominal and other lymph glands .. | 18 | ,, |
| Liver .. .. .. .. .. | 13 | ,, |
| Lungs (away from main growth) .. | 9 | ,, |
| Adrenals .. .. .. .. .. | 9 | ,, |
| Bones .. .. .. .. .. | 4 | ,, |
| Kidneys .. .. .. .. .. | 4 | ,, |
| Brain .. .. .. .. .. | 3 | ,, |
| Pancreas .. .. .. .. .. | 2 | ,, |
| Ovaries, skin, thyroid, etc. .. .. | 1 | case. |

In some cases the first symptoms noted have been due to the metastatic deposits, and not to the primary growth itself. Thus a woman of 36, admitted for jaundice, abdominal pain and vomiting, was found at post-mortem to have massive deposits in the pancreas and liver, and an insignificant bronchial primary growth which was unsuspected during life. Similarly, sciatic pain and a thigh tumour were the first symptoms in a man of 54, who later suffered a spontaneous fracture of the humerus, both due to secondary growth from a small primary in the left bronchus. In other cases mental symptoms developing in the course of atypical "pneumonias" served to strengthen the clinical suspicion of malignant lung disease, while a further case in the writer's recent experience (not included in the present series) presented itself primarily as one of acute mania; at post-mortem the brain was found riddled with secondaries from a small bronchial tumour which had given rise to no symptoms whatever during life. It is probable that brain secondaries occur more frequently than is indicated in the above table, since this organ was examined in less than half the total number of cases. It will be seen that deposits in the adrenals are relatively common, a fact which is no doubt due to their close proximity to the diaphragmatic lymphatics. Seven of the 9 cases showed bilateral involvement; in 3 of these the more rapid clinical course with marked asthenia and skin pigmentation was undoubtedly due to malignant destruction of these glands.

LABORATORY DIAGNOSIS.—As this disease is rapidly progressive, and soon passes beyond the stage where treatment is helpful, it is obvious that the earliest possible diagnosis is essential if any treatment is to be of benefit. The histology of invaded lymph glands or secondary skin nodules is, of course, merely of academic interest, since by this time the case is beyond hope. Examination of pleural or other effusions for malignant cells is rarely helpful, partly because the fluid collection usually occurs late in the disease, partly since malignant cells cannot as a rule be identified with certainty by ordinary staining methods. There are two other procedures, however, which are much more likely to afford early information. One is the microscopic examination of a fragment of tumour tissue removed by bronchoscopy. In four recent cases we were able thus to identify the type of growth and clinch the diagnosis at a comparatively early date. The other is the microscopy of the blood-stained sputum, which is generally an early sign of bronchial carcinoma. Such sputum, making its appearance as soon as ulceration occurs in the affected bronchus, frequently contains malignant cells, but very rarely fragments large enough for sectioning. In the search for these cells ordinary staining methods have not been very helpful, but specimens treated by the wet-film method of Dudgeon and Wrigley give excellent preparations in which they may often be identified. In a valuable recent paper (*Jour. Laryng. and Otol.*, 1935, 50, 752) these workers reported that in 68 per cent. of 38 proved cases of carcinoma of lung or larynx it was possible by this method to establish the diagnosis from examination of the sputum, and in the majority of cases to distinguish the histological type of growth present. We have lately had an opportunity of applying the method in four suspected cases, in two of which typical "oat-cells" were detected in the films; in one of these the diagnosis was further confirmed by histology of a fragment removed by the bronchoscope. There is no doubt that the method represents a great advance and should be widely known, for unless the diagnosis of bronchial carcinoma is established at the earliest possible moment surgical cure is manifestly impossible, and even palliative treatment has slender hope of success.

